# The effects of family physician-contracted service on health-related quality of life and equity in health in China

**DOI:** 10.1186/s12939-020-01348-4

**Published:** 2021-01-06

**Authors:** Sha Lai, Li Lu, Zhongliang Zhou, Chi Shen, Xiaowei Yang, Yaxin Zhao, Xiaolong Zhang

**Affiliations:** 1 School of Public Policy and Administration, Xi’an Jiaotong University, No. 28 West Xianning Road, Xi’an, 710048 Shaanxi China; 2 Team IETO, Bordeaux Population Health Research Center, UMR U1219, INSERM, Université de Bordeaux, Bordeaux, France

**Keywords:** Family physician, Health-related quality of life, Equity in health, China

## Abstract

**Background:**

Family physician-contracted service (FPCs) has been recently implemented in Chinese primary care settings. This study was aimed at measuring the effects of FPCs on residents’ health-related quality of life (HRQoL) and equity in health among the Chinese population.

**Methods:**

The study data was drawn from the 2018 household health survey (Shaanxi Province, China) using multistage, stratified cluster random sampling. We measured HRQoL using EQ-5D-3L based on the Chinese-specific time trade-off values set. Coarsened exact matching (CEM) technique was used to control for confounding factors between residents with and without a contracted family physician. The concentration index (C) was calculated to measure equity in health.

**Results:**

Individuals with a contracted family physician had significantly higher HRQoL than those without, after data matching (0.9355 vs. 0.8995; *P* <  0.001). Additionally, the inequity in HRQoL among respondents with a contracted family physician was significantly lower than those without a contracted family physician (Cs of EQ-5D utility score: 0.0084 vs. 0.0263; *p* <  0.001).

**Conclusions:**

This study highlights the positive effects of FPCs on HRQoL and socioeconomic-related equity in HRQoL. Future efforts should prioritize the economically and educationally disadvantaged groups, the expansion of service coverage, and the competency of family physician teams to further enhance health outcome and equity in health.

## Background

Family physician (also known as the general practitioner) system is considered as the core of primary care and has gained rapid ground worldwide [1–3]. Primary care services provided by family physician enhance the continuity and coordination of care, reduce the inappropriate use of specialty services, and improve a population’s health [4–6]. The Chinese government has explored and established a hierarchical medical system to suit the national conditions in recent years [7]. The hierarchical medical system emphasizes the importance of a clear division of health care roles and responsibilities for different levels of hospitals, and highlights the significance of taking strategies to encourage the utilization of primary care [8]. The implementation of the family physician-contracted service (FPCs) is one of the key actions, which improve people’s access to primary health care, minimize the unfair distribution of medical resources and improve the health of community residents [9–11].

Family physician-contracted services were officially launched throughout China in June 2016 after pilot projects in 200 selected areas [12]. Family physician-contracted services target the entire population, focusing on the elderly, pregnant women, children, people with disabilities, and patients with hypertension, diabetes, tuberculosis and other chronic diseases. The public is being encouraged and guided to contract with nearby family physician teams who are responsible for the provision of proactive, continuous, and comprehensive health care [12]. Family physician-contracted services are being explored and implemented in various regions of China. For example, in Shaanxi province (part of Northwest China), the FPCs—fully launched in 2016—provides ‘free service package + paid service package’ and its operational cost is partly subsidized from the regional funds of medical insurance and basic public health program [13]. The free service package was designed to provide basic public health services: such as establishing and managing personal health records, health education, health literacy promotion, and primary care management of chronic diseases. The paid service package includes basic medical services and personalized health management services. The residents who are contracted with a family physician can enjoy free or low-cost healthcare services from the family physician team, which include family physicians, nurses, public health practitioners.

The effects of FPCs have been widely reported in previous studies. Family physician-contracted services can improve the continuity, comprehensiveness, and coordination of primary care services [14–16]. This service also helps individuals with chronic diseases to improve their health-related awareness, self-management behaviors, and treatment compliance [17, 18]. For example, a population-based retrospective cohort study in Canada suggested that family physician care can reduce hospitalizations in elderly people with diabetes [19]. Another randomized controlled trial study in Norway identified that in addition to geriatrician, clinical assessments and medication reviews from collaborative team including family physicians can improve health-related quality of life (HRQoL) in older patients exposed to polypharmacy [20]. However, one Iranian study indicated that family physician program had a positive effect on the proximal health indicators in maternal and child health (e.g., birth weight), but no substantial effect on mortality [21]. One study from South Africa did not find a positive association between the supply of family physician and health indicators (i.e., maternal mortality, perinatal mortality and under-five mortality) [22]. The inconsistencies among existing studies could be partly attributed to different health indicators that were used.

Existing studies concerning the effects of FPCs on health in China are mainly limited on improving health outcomes in patients with chronic diseases. For example, previous studies have shown that FPCs have positive effects on hypertension control rates among patients with hypertension [18, 23]. Unfortunately, in China, whether family physician-contracting services could improve HRQoL and health equity in the general population has not been extensively studied.

Therefore, we conducted this study using the data from a household health survey in Shaanxi Province, China, with the aim to (1) explore whether being contracted with family physician services can improve HRQoL, (2) investigate whether the inequity in HRQoL would be lessened under the implementation of FPCs, which might provide implications for policymakers in terms of advancing family practice contract services in China. Based on previous research [15, 24–26], we hypothesize that FPCs would help improve community residents’ HRQoL and lessen the inequity in HRQoL.

## Methods

### Data sources

Data were derived from the 2018 cross-sectional household health survey that was conducted in three districts and two counties in Shaanxi Province, an area about 205,800 km^2^ in northwest China with a population of 37.9 million.

Participants were recruited by multistage, stratified cluster random sampling. In short, out of 5 districts or counties, 25 sub-districts or townships were randomly selected. Out of those sub-districts, we randomly selected 50 communities or villages (two for each sub-district or township). All members from 3000 households including 7819 individuals were collected. Only participants aged 15 years and above were included in this study, since children who were below 15 years could not answer several questions regarding socioeconomic characteristics and health status. Finally, 6503 individuals were included in the analyses.

### Measures

Pre-trained interviewers conducted face-to-face interviews by structured questionnaire, and collected the following information: sociodemographic characteristics, economic characteristics, health status, health services coverage, and health services utilization.

Sociodemographic and economic characteristics include the following information: sex, age group (15–44 years/45–59 years/60 years and above), educational level (primary and below/junior middle school/senior middle school/ college and above), marital status (unmarried/married/others), employment status (unemployed/employed), commercial medical insurance (with/without), minimum travel time to the nearest health-care facility (within 15 min/more than 15 min), basic medical insurance (Urban Employee Basic Medical Insurance [UEBMI]/Urban-Rural Resident Basic Medical Insurance [URRBMI]), chronic conditions (yes/no), and residential areas (urban or rural areas). The household consumption expenditure per equivalent adult was used as a proxy measure of economic status. We divided the household consumption expenditure per equivalent adult into five groups, the first quintile represents the poorest economic group (i.e., the lowest 20%) while the fifth quintile represents the wealthiest economic group (i.e., the highest 20%).

We inquired whether responders had contracted with a family physician by asking “Are you contracted with a family physician?”, which they could respond with “yes” (scored as 1) and “no” or “I’ve heard nothing of this services” (scored as 0).

Health-related quality of life (HRQoL) combines physical and mental health into a summary score, which ranges from 0 (death) to 1 (perfect health). Notably, certain scores can also measure states worse than death [27], such as an index score of EQ-5D ranges − 0.59 (worst possible health state) to 1 (best possible health states) according UK tariffs, and by construction, the value of 0 is equal to death and negative values represent HRQoL worse than being dead [28]. In our study, HRQoL was measured by the health utility values for the validated Chinese version of the EuroQol five-dimensional questionnaire-three-level version (EQ-5D-3L) [29, 30]. The questionnaire includes five questions and consists of five dimensions, i.e., responder’s mobility, self-care, usual activities, pain or discomfort, and anxiety or depression; each dimension has three response levels (1 = no problems, 2 = moderate problems and 3 = extreme problems). We employed the Chinese time trade-off values for EQ-5D-3L to measure the utility values of the EQ-5D-3L [31], which has also been widely used in other studies [32, 33]. The overall utility values of EQ-5D-3L ranges from − 0.149 (having extreme problems) to 1 (no problems).

### Statistical analyses

#### Matching method

##### Coarsened exact matching (CEM)

The pretreatment covariates differ between the groups with and without FPCs for the observational data, the Coarsened Exact Matching (CEM) technique is designed to improve the estimation of causal effects via a powerfully matching method to keep a better balance of distributions of the covariates between groups, and thereby reducing the bias [34]. The idea of CEM is to temporarily coarsen each variable into substantively meaningful groups, match on these coarsened data exactly, and then only retain the original (uncoarsened) values of the matched data [35]. We applied the CEM method to control confounding variables between the two groups to investigate whether a contracted family physician could improve HRQoL and promote health equity. The detail of technique is well described in detail in previous literatures [34, 35]. The STATA *cem* command was used to perform the CEM algorithm [35].

There is a comprehensive imbalance measure *L*_1_ statistic, which was used to check the overall imbalance. The calculation for *L*_1_ is as follows: first, we coarsened the covariates into bins, then cross-tabulated the discretized variables as *X*_1_ × … × *X*_*k*_ for the groups with and without FPCs separately, and recorded the k-dimensional relative frequencies for the group with FPCs ${f}_{e_1\dots {e}_k}$ and for the control ${g}_{e_1\dots {e}_k}$ units. Finally, the absolute difference over all the cell values is defined as the measure of imbalance *L*_1_ :[35].

1 $$ {L}_1\left(f,g\right)=\frac{1}{2}{\sum}_{e_1\dots {e}_k}\left|{f}_{e_1\dots {e}_k}-{g}_{e_1\dots {e}_k}\right| $$

*L*_1_ ranges from 0 (perfect global balance) to 1 (maximal imbalance). A substantial reduction in *L*_1_ means a good matching performance. In this study, we focused on matching the following covariates: sex, age, chronic conditions, economic status, educational level, employment status, marital status, medical insurance, spatial accessibility of health-care facility, and residential areas.

#### Tobit regression models

##### (a) Concentration index (C)

We measured the degree of socioeconomic-related inequality in HRQoL between the two groups in our study using the concentration index (C), which is a widely used parameter [36, 37]. The C takes values in the range [− 1, 1]; a value of 0 means perfect equality, a positive value signifies pro-rich bias while a negative value signifies pro-poor bias. The C formula is

2 $$ \mathrm{C}=\frac{2}{\mu}\mathit{\operatorname{cov}}\left({y}_i,{r}_i\right) $$

where *y* is the health variable (i.e., the utility values of the EQ-5D in this study), *μ* is mean of the EQ-5D utility value, *r*_*i*_ (rang [0, 1]) is the fractional rank of the *i* th individual in the economic distribution.

##### (b) Decomposition analysis for C.

The decomposition analysis was based on a regression model that decomposes C into its contributing factors. Tobit regression was applied to calculate the partial effects of regressors, owing to the outcome variables (i.e., the utility values of EQ-5D) which were limited variables [38]. Health (*y*) is modelled as follows: [36].

3 $$ {y}_i={\sum}_k{\beta}_k{x}_{ki}+{\varepsilon}_i $$

*β*_*k*_ are the partial effects, *ε* is the error term, while *x*_*k*_ are the means of explanatory variables. C(*y*) can be decomposed as: [36].

4 $$ \mathrm{C}={\sum}_k\left(\frac{\beta_k{\overline{x}}_k}{\mu}\right){C}_k+\frac{GC_{\varepsilon }}{\mu } $$

*C*_*k*_ is the concentration index of explanatory variables and *GC*_*ε*_ is the generalized concentration index of *ε*. The deterministic component is ${\sum}_k\left(\frac{\beta_k{\overline{x}}_k}{\mu}\right){C}_k$, while the residual component is $\frac{GC_{\varepsilon }}{\mu }$. The contribution of each factor depends on its impact on health and the degree of unequal distribution across the economic gradient. The percentage contribution of each regressor (100*Q*_*k*_/*C*) was also calculated, and it can take both positive and negative values. We further calculated the age-sex adjusted C (also known as the horizontal inequity index)—which represents the potentially avoidable inequality—by subtracting the contributions of age and gender from the total C [39].

Standard errors were adjusted for clustering at the family level for all models. Statistical analyses were conducted using the STATA version 14.0 (Stata Corporation, College Station, Texas, USA) with the significance level as a *P* <  0.05 (two-tailed).

## Results

### Demographic characteristics of responders and matching performances

Table 1 represents basic sociodemographic and economic characteristics of the respondents before and after data matching. A total of 6503 respondents were included, and data from 4612 individuals were further analyzed after data matching. Prior to data matching, the proportion of respondents aged 60 and above, having chronic conditions, with middle and below economic status, with junior middle school and below education, having Urban-Rural Resident Basic Medical Insurance, and living in rural region in group with FPCs was greater than group without FPCs. After matching using CEM method, covariates imbalances were eliminated between the two groups. Table 2 shows *L*_1_ statistics of each variable, and multivariate *L*_1_ statistics before and after data matching. Consistently, in the two groups, *L*_1_ values of each variable decreased and were close to zero after data matching.

**Table 1:** Characteristics of respondents among those with and without a contracted family physician before and after data matching

		Before Matching					After Matching				
Variables		With contracted family physician		Without contracted family physician		*P*^a^	With contracted family physician		Without contracted family physician		*P*^a^
		N	%	N	%		N	%	N	%	
Sex	Male^b^	1690	47.53	1425	48.35	0.505	1093	44.98	981	44.98	1.000
	Female	1866	52.47	1522	51.65		1337	55.02	1201	55.02	
Age group (year)	15–44^b^	1013	28.49	1085	36.82	< 0.001	724	29.79	650	29.79	1.000
	45–59	1220	34.31	1010	34.27		795	32.72	714	32.72	
	60 and above	1323	37.20	852	28.91		911	37.49	818	37.49	
Chronic conditions	No^b^	2226	62.60	2031	68.92	< 0.001	1616	66.50	1451	66.50	1.000
	Yes	1330	37.40	916	31.08		814	33.50	731	33.50	
Economic status	Poorest^b^	746	20.98	559	18.97	< 0.001	544	22.39	488	22.39	1.000
	Poorer	803	22.58	495	16.80		592	24.36	532	24.36	
	Middle	755	21.23	544	18.46		457	18.81	410	18.81	
	Richer	754	21.20	730	24.77		496	20.41	445	20.41	
	Richest	498	14.00	619	21.00		341	14.03	306	14.03	
Educational level	Primary or below^b^	1273	35.80	681	23.11	< 0.001	887	36.50	796	36.50	1.000
	Junior middle school	1345	37.82	1061	36.00		967	39.79	868	39.79	
	Senior middle school	600	16.87	687	23.31		345	14.20	310	14.20	
	College or above	338	9.51	518	17.58		231	9.51	207	9.51	
Marital status	Unmarried^b^	248	6.97	248	8.42	0.040	107	4.40	96	4.40	1.000
	Married	2989	84.06	2465	83.64		2181	89.75	1958	89.75	
	Other	319	8.97	234	7.94		142	5.84	128	5.84	
Employment status	Employed	1953	54.92	1694	57.48	0.038	1383	56.91	1242	56.91	1.000
	Unemployed^b^	1603	45.08	1253	42.52		1047	43.09	940	43.09	
Basic medical insurance	UEBMI^b^	549	15.44	901	30.57	< 0.001	437	17.98	392	17.98	1.000
	URRBMI	3007	84.56	2046	69.43		1993	82.02	1790	82.02	
Commercial insurance	No^b^	3096	87.06	2559	86.83	0.784	2255	92.80	2025	92.80	1.000
	Yes	460	12.94	388	13.17		175	7.20	157	7.20	
Minimum travel time to the nearest health-care facility, N (%)	Within 15 min	3328	93.59	2751	93.35	0.697	2351	96.75	2111	96.75	1.000
	More than 15 min^b^	228	6.41	196	6.65		79	3.25	71	3.25	
Residential areas	Urban	1507	42.38	2499	84.80	< 0.001	1237	50.91	1111	50.91	1.000
	Rural^b^	2049	57.62	448	15.20		1193	49.09	1071	49.09	
Total		3556		2947			2430		2182		

*UEBMI* Urban Employee Basic Medical Insurance, *URRBMI* Urban-Rural Resident Basic Medical Insurance

^a^Chi-square test is used for balance checking between two groups

^b^Reference levels in the Tobit regression

**Table 2:** The L_1_ measure of imbalance between two groups before and after Coarsened Exact Matching

Variables	Before Matching		After Matching	
	*L*_1_ (Mean)	*L*_1_ (SE)	*L*_1_ (Mean)	*L*_1_ (SE)
Sex	0.0083	0.0083	2.6 × 10^−15^	2.9 × 10^−15^
Age group	0.0833	0.1662	3.8 × 10^− 15^	−7.1 × 10^− 15^
Chronic conditions	0.0632	0.0632	2.2 × 10^− 15^	2.8 × 10^− 15^
Economic status	0.1057	−0.2737	2.7 × 10^− 15^	−3.1 × 10^− 15^
Educational level	0.1451	−0.3527	3.2 × 10^− 15^	−5.3 × 10^− 15^
Marital status	0.0144	0.0247	3.7 × 10^−16^	−4.4 × 10^− 15^
Employment status	0.0256	0.0256	2.8 × 10^−15^	1.3 × 10^− 15^
Basic medical insurance	0.1514	0.1514	7.9 × 10^−16^	−2.4 × 10^−15^
Commercial insurance	0.0023	−0.0023	6.2 × 10^−16^	4.7 × 10^−16^
Minimum travel time to the nearest health-care facility	0.0024	−0.0024	1.2 × 10^−16^	1.1 × 10^−15^
Residential areas	0.4242	3.3935	3.1 × 10^−15^	5.5 × 10^−14^
Multivariate L_1_	0.5648		3.6 × 10^−15^	
Total, N	6503		4566	

Note: The overall imbalance is given by *L*_1_ statistic, introduced in Iacus, King, and Porro (2008) as a comprehensive measure of global imbalance. *L*_1_ reported the *L*_1*j*_ measure, which is *L*_1_ computed for the *j*_*th*_ variable separated. The mean was labeled in parentheses reported the difference in means

### Description of EQ-5D and its concentration index

The utility values of EQ-5D and its each dimension are presented in Table 3. After data matching, the mean EQ-5D utility values between respondents with FPCs were 0.9355 (95% CI: 0.9302–0.9409), and without FPCs were 0.8995 (95% CI: 0.8926–0.9063). The Cs of EQ-5D score for the respondents with a contracted family physician was significantly lower than those without a contracted family physician (0.0084, 95% CI: 0.0047–0.0122 vs. 0.0263, 95% CI: 0.0187–0.0340; *P* <  0.001), suggesting that the pro-rich bias was slightly reduced by contracting family physician services.

**Table 3:** Description of EQ-5D health state and economic-related inequality in EQ-5D scores between respondents with and without contracted family physician

		Before matching					After matching				
		With contracted family physician		Without contracted family physician		*p*	With contracted family physician		Without contracted family physician		*p*
		Mean	95% CI	Mean	95% CI		Mean	95% CI	Mean	95% CI	
Mobility	Moderate problem	0.0928	(0.0837, 0.1028)	0.0926	(0.0827, 0.1037)	< 0.001	0.0947	(0.0836, 0.1070)	0.1498	(0.1354, 0.1654)	< 0.001
	Extreme problem	0.0076	(0.0052, 0.0111)	0.0078	(0.0052, 0.0117)	0.249	0.0058	(0.0034, 0.0097)	0.0102	(0.0067, 0.0154)	0.150
Selfcare	Moderate problem	0.0397	(0.0337, 0.0466)	0.0336	(0.0277, 0.0407)	0.434	0.0383	(0.0313, 0.0467)	0.0549	(0.0461, 0.0653)	0.065
	Extreme problem	0.0096	(0.0068, 0.0134)	0.0088	(0.0060, 0.0129)	0.422	0.0082	(0.0053, 0.0127)	0.0141	(0.0099, 0.0200)	0.165
Activity	Moderate problem	0.0560	(0.0489, 0.0640)	0.0506	(0.0432, 0.0591)	0.117	0.0556	(0.0471, 0.0654)	0.0755	(0.0651, 0.0874)	0.045
	Extreme problem	0.0200	(0.0159, 0.0251)	0.0227	(0.0179, 0.0288)	0.002	0.0181	(0.0135, 0.0242)	0.0431	(0.0353, 0.0525)	< 0.001
Pain	Moderate problem	0.1997	(0.1868, 0.2131)	0.2029	(0.1888, 0.2178)	< 0.001	0.2008	(0.1854, 0.2172)	0.2707	(0.2525, 0.2898)	< 0.001
	Extreme problem	0.0087	(0.0061, 0.0124)	0.0143	(0.0105, 0.0192)	0.001	0.0082	(0.0053, 0.0127)	0.0318	(0.0252, 0.0400)	< 0.001
Anxiety	Moderate problem	0.0906	(0.0815, 0.1004)	0.0882	(0.0785, 0.0990)	0.102	0.0897	(0.0790, 0.1017)	0.1196	(0.1067, 0.1340)	0.016
	Extreme problem	0.0051	(0.0032, 0.0080)	0.0071	(0.0046, 0.0109)	0.178	0.0041	(0.0022, 0.0076)	0.0059	(0.0034, 0.0101)	0.555
EQ-5D scores		0.9341	(0.9295, 0.9386)	0.9322	(0.9272, 0.9372)	< 0.001	0.9355	(0.9302, 0.9409)	0.8995	(0.8926, 0.9063)	< 0.001
C_EQ-5D_		0.0084	(0.0052, 0.0116)	0.0137	(0.0114, 0.0161)	0.029	0.0084	(0.0047, 0.0122)	0.0263	(0.0187, 0.0340)	< 0.001

*Note: 95% CI:* 95% Confidence Interval, *C:* Concentration Index

Statistically differences (*P* <  0.05) in each dimension of EQ-5D between two groups based on Multinomial logistic regression (“No problem” was set as the base outcome); statistically differences (*P* <  0.05) in utility values of EQ-5D between two groups based on Tobit regressions; all regression adjusted for sex, age group, chronic conditions, economic status, education level, marital status, working status, basic medical insurance, commercial medical insurance, minimum travel time to the nearest health-care facility and residential areas

### Decomposition of inequality of HRQoL

Table 4 presents the decomposition results of Cs of the overall EQ-5D scores. The partial effects, absolute contribution and percentage contribution of each determinant to the inequality of the overall EQ-5D score are presented.

**Table 4:** Decomposition of Concentration Index of the EQ-5D Scores between respondents with and without contracted family physician

		Before matching						After matching					
		With contracted family physician			Without contracted family physician			With contracted family physician			Without contracted family physician		
Variables		*dy*/*dx*	con.	%con.	*dy*/*dx*	con.	%con.	*dy*/*dx*	con.	%con.	*dy*/*dx*	con.	%con.
Sex	Female	0.009	< 0.001	0.150	− 0.034*	< 0.001	− 0.453	0.017	< 0.001	− 0.486	− 0.048	< 0.001	0.633
Age group	45–59 years	− 0.118***	− 0.002	− 26.518	− 0.077***	− 0.001	−4.627	− 0.133***	− 0.002	−29.125	− 0.068	− 0.001	− 5.577
	60 years and above	− 0.160***	0.003	40.722	− 0.131***	0.002	17.125	−0.195***	0.008	90.490	−0.130*	0.005	20.808
Chronic conditions	Yes	−0.211***	−0.001	−14.140	− 0.184***	0.001	6.144	−0.195***	− 0.001	−9.299	−0.169***	< 0.001	− 1.833
Economic status	Poorer	0.026	−0.002	−26.701	0.031	−0.003	− 18.485	0.007	−0.001	−6.252	0.041	−0.003	−13.158
	Middle	0.062**	0.001	14.007	0.044	−0.001	−6.334	0.042	0.001	12.373	0.066	0.002	6.493
	Richer	0.056*	0.006	76.268	0.100***	0.009	64.278	0.001	< 0.001	1.964	0.091*	0.011	40.612
	Richest	0.062*	0.008	95.330	0.102***	0.018	132.104	0.045	0.006	68.097	0.115**	0.015	58.622
Educational level	Junior middle school	0.119***	0.001	12.989	0.105***	−0.005	−34.065	0.136***	0.001	10.376	0.092**	0.001	2.664
	Senior middle school	0.096***	0.003	40.052	0.157***	0.006	45.973	0.121***	0.005	63.600	0.148***	0.007	25.668
	College or above	0.182 ***	0.007	85.576	0.164***	0.012	87.094	0.125*	0.007	79.435	0.126**	0.007	27.006
Marital status	Married	−0.036	−0.001	−10.310	−0.039	−0.001	−5.762	− 0.159*	− 0.003	−41.147	− 0.025	−0.001	−2.211
	Other	−0.093 *	0.001	8.458	−0.150***	0.002	15.067	−0.217**	0.003	39.375	−0.167*	0.003	11.104
Employment status	Employed	0.071***	< 0.001	2.135	0.043*	−0.001	−5.354	0.056**	< 0.001	−0.691	0.040	< 0.001	−0.191
Basic Medical Insurance	URRBMI	−0.020	0.002	22.316	0.026	−0.004	−25.883	−0.028	0.003	41.146	−0.006	0.001	2.907
Commercial insurance	Yes	0.069**	0.002	19.545	−0.009	< 0.001	−1.125	0.064	0.001	16.933	0.012	< 0.001	1.077
Minimum travel time to the nearest health-care facility	Within 15 min	0.066*	0.001	17.622	0.065	0.001	5.239	0.049	0.001	11.538	0.173***	0.003	13.210
Residential areas	Urban	−0.019	−0.001	−14.754	0.075***	0.003	24.738	−0.019	−0.002	−20.454	0.071**	0.007	26.298
C			0.008			0.014			0.008			0.026	
Age-sex C			0.007			0.012			0.003			0.022	

Note: *dy*/*dx*: Partial effect in Tobit regression model; Con.: The absolute contribution of each determinant (${Q}_k={\beta}_k\frac{{\overline{x}}_k}{\mu }{C}_k$); %con.: The percentage contribution of each determinant to the total concentration index (100*Q*_*k*_/*C*)

*p < 0.05, **p < 0.01, ***p < 0.001

Partial effect estimates indicated that among respondents with a contracted family physician, those aged 45 years and above, having chronic conditions, and married, divorced and widowed status were more likely to have lower overall HRQoL. Among those who did not contract with a family physician, the following factors were negatively associated with the overall HRQoL: aged 60 years and above, presence of chronic conditions, low economic status and educational level, divorced and widowed status, living in rural areas and far from health-care facilities.

A positive contribution to inequality means that the relevant determinant increases pro-rich inequality and vice versa. For those with a contracted family physician, the top three categories of residents with the greatest inequality in HRQoL were: aged 60 years and above (90.49%), having at least college-level education (79.44%) and richest economic status (68.10%). For those without a contracted family physician, the following factors had the largest positive contributions in explaining the inequality of HRQoL: richest economic status (58.62%), richer economic status (40.61%), and having at least college-level education (27.01%). The age-sex adjusted Cs in HRQoL among those with a contracted family physician was lower (0.003) than those without (0.022).

## Discussion

Benefiting from the CEM technique and a representative dataset (the 2018 household health survey in Shaanxi Province, northwest China), our study found that individuals with a contracted family physician had significantly better HRQoL that was measured with EQ-5D-3L than those without. Moreover, it also suggested that the inequities in HRQoL were lower among those who contracted with a family physician than those who did not.

Family physician services are favorably helpful in promoting HRQoL [20, 40]—which was reconfirmed in this study showing that the EQ-5D-3L utility value among individuals who contracted with family physician services was greater than those who did not (*P* <  0.001). HRQoL is a generic outcome measure to assess the effectiveness of interventions and a way of reflecting one’s subjective perceptions and experiences [41]. Impaired HRQoL denotes perceived difficulties and functional limitations in daily life caused by illness [27]. The results also suggested that individuals contracted with a family physician were less likely to have functional limitations in moderate mobility, activity, pain and moderate anxiety. On the one hand, the health care services delivered by the family physicians include medicine and physical therapies, rehabilitation services, health counseling and the referral services. These proactive, continuous and comprehensive health care services could help enhancing the physical ability and mental health of contracted patients, such as relieving pain, improving mobility and activity, relieving depression and anxiety. On the other hand, the long-term cooperative relationships between contracted family physicians and residents promotes trust in primary care physicians among residents, which were identified to be associated with lower depression and anxiety in patients [42]. FPCs is a comprehensive strategy that strives to ensure equitable access, continuity of care, coordination and comprehensiveness of care in primary healthcare. Previous literature has identified the remarkable effects of FPCs in improving the quality and utilization of primary healthcare services [16, 43], which are highly correlated with better health outcomes, including total and specific mortality, life expectancy, and other health outcome indices, e.g., self-rated health, HRQoL [24].

Our study found that the pro-rich bias was slightly reduced by family physician-contracted services; age-sex adjusted Cs in HRQoL were 0.003 among the group with FPCs, and 0.022 among the group without FPCs—indicating the role of FPCs in reducing health disparities. Striking differences in health outcome still exist within and between populations, so equity is an important goal in health sectors. The primary healthcare system is important to promote equity in access and health outcome of a population, yet primary healthcare services are not always readily accessible for some populations in China [44]. The FPCs in China established a long-term contracted relationship between residents and family physicians. They provide comprehensive clinical care and health management services to the contracted populations**,** and promote information exchange; thus helping to improve the accessibility of primary health services [45]. The cost of FPCs is partially subsidized by public finances, which may reduce barriers to healthcare utilization for disadvantaged groups and thereby improving their health status [46].

From a policy perspective, improvement in health equity needs a broader focus that addresses socioeconomic determinants of health and health equity. The current FPCs gives the high-need populations, such as the elderly, pregnant women, children, people with disability, and people with chronic diseases, the top priority to primary healthcare services in our sample areas. Our findings suggested that lower socioeconomic status, i.e., lower educational level, unemployment, poor spatial accessibility, and living in rural areas were associated with lower HRQoL. In addition, economic status and educational level were the main sources of inequities in HRQoL among both individuals with and without FPCs. Therefore, authorities need to pay more attention to the economically and educationally disadvantaged groups to further enhance health equity.

Improving health status and the longstanding inequity of health and healthcare still remain as challenges nevertheless there was progress in implementing FPCs and primary healthcare services [10]. Firstly, further efforts to improve people’s awareness and willingness to contract with a family physician are required. In Shaanxi province, the signing contract rate among general residents was 54.2% (according to our full sample data) in 2018. Previous studies showed that the signing contract rates varied greatly in different Chinese regions (30–60%) [47–49]. Secondly, the family physician service team should be expanded further to include other allied health professionals such as counselling psychologists, health managers, pharmacists and social workers; this would enhance the capability of the FPCs team to provide better health services [50]. General practitioners, community nurses and public health practitioners are the main components of the current FPCs team, which may not be capable of meeting residents’ multifaceted healthcare needs, such as psychological counseling and care services which as we found no significant effects in HRQoL in the dimension of self-care and extreme anxiety. Well-implemented primary healthcare services tend to bring benefits to the most vulnerable in the communities as well as to those with complex healthcare needs [44].

One of our study merits is that we examined the effects of FPCs on health and performed the CEM technique to guarantee better balance of covariate distribution between individuals with and without FPCs. The second is that we used the EQ-5D-3L instrument that was validated among the Chinese general population [30] as a health outcome measure to assess the effect of FPCs on our sample. Some limitations should be noted. Firstly, some unobservable or unmatched factors, such as health literacy and beliefs, may have potential effects on the results. Secondly, the results should be explained as associations rather than causal effects as we used cross-sectional data, thus further longitudinal studies should be conducted. In addition, the severe ceiling effect of EQ-5D-3L cannot be fully eliminated when measuring HRQoL among the general population. Specifically, although 71.7% of the residents in our study rated themselves as in full health, it was slightly smaller than results in another Chinese study [51]. Lastly, the samples were obtained from one province, and there were heterogeneities regarding FPCs policies and socioeconomic environments between regions, which might limit the study generalizability.

## Conclusion

This study sheds light on the positive effects of FPCs on the HRQoL and socioeconomic-related equity by comparing the magnitude of inequalities in HRQoL between the individuals with and without FPCs. To further enhance health equity, authorities should pay more attention to the economically and educationally disadvantaged individuals. Our study provided evidence for policy-makers and healthcare institutions to promote and implement FPCs, extend the service coverage of family physicians and enhance the professional competency of family physicians service team.

## Data Availability

The data used in this study belong to the Health Commission of Shaanxi Province and contain the personal information (e.g., name, personal communication information, property status, health condition, physical defect etc.) of participants. The authors were involved in data collection. Due to the sensitive nature of these data and restrictions imposed by the Health Commission of Shaanxi Province, the authors cannot make these data publicly available. Other researchers who want to use the data may submit requests for data access to the Health Commission of Shaanxi Province at sxwjwwz@126.com.

## Abbreviations

FPCs: Family physician-contracted service
HRQoL: Health-related quality of life
EQ-5D-3L: EuroQol five-dimensional questionnaire-three-level version
UEBMI: Urban Employee Basic Medical Insurance
URRBMI: Urban-Rural Resident Basic Medical Insurance
CEM: Coarsened Exact Matching
C: Concentration index
